# The Molecular Characterization and Functional Analysis of *Pomacea canaliculata* Boule: A Central Player in Spermatogenesis and Male Fertility

**DOI:** 10.3390/biology15070554

**Published:** 2026-03-30

**Authors:** Haotian Gu, Tianshu Zhang, Yongda Yuan, Haiyuan Teng

**Affiliations:** 1 Shanghai Key Laboratory of Protected Horticultural Technology, Eco-Environmental Protection Research Institute, Shanghai Academy of Agricultural Sciences, Shanghai 201403, China; guhaotian@saas.sh.cn (H.G.);; 2 Shanghai Engineering Research Centre of Low-Carbon Agriculture (SERCLA), Shanghai 201415, China

**Keywords:** spermatogenesis, boule, *Pomacea canaliculata*, male fertility, RNAi

## Abstract

*Boule* is the ancestral member of the *Deleted in Azoospermia* (*DAZ*) family and is pivotal for gametogenesis and male fertility in most animals. However, there is a dearth of information on molluscan *boule*. Here, we identified a counterpart (*Pcbol*) from the genome of *Pomacea canaliculata*, which has emerged as a cosmopolitan alien species and notorious pest that causes devastating damage to aquatic biodiversity, freshwater ecosystems and crop production in invaded ranges. This study aimed to investigate the biological roles of *Pcbol* in male reproduction and to decipher the molecular mechanisms underpinning its modulation via dsRNA-delivered RNA interference (RNAi). The bioinformatic analysis showed that the *Pcbol* genomic sequence is 12,934 nt in length, harboring an open reading frame of 294 nt that encodes 97 aa residues, with an RRM domain evolutionarily conserved among molluscan orthologues. Spatiotemporal expression profiling indicated the predominant abundance of Pcbol in adult males and testis tissues. dsPcbol, injected at a dose of 4 μg/per snail for 5 days, yielded optimal silencing at both transcript and translation levels of Pcbol, as revealed by qRT-PCR and Western blotting. Immunofluorescence echoed a pronounced reduction in Pcbol signal intensity following RNAi. In addition to the arrested reproductive gland phenotype, the number of sperm cells substantially dwindled upon dsPcbol treatment relative to the dsGFP control. In biochemical and fecundity assays, *Pcbol* depletion triggered a significant decrease in Te/SP/Arg content and suppressed the number of deposited eggs and hatchability. Furthermore, spermatogenic genes like *CDC25/TSSK1/SPATA17/DDX4/Dmrt2/Sox2/Kelch10/SPO11* displayed considerable downregulation post *Pcbol* silencing, with molecular docking predicting a strong affinity between CDC25 and Pcbol. These molecular modules may interact with Pcbol to mediate knockdown effects on spermatogenesis dysfunction. Collectively, our findings not only confirmed that *boule* was indispensable for spermatogenesis and male fertility in a mollusk, but also highlighted the *Pcbol*-based male sterile technique (MST), which can be incorporated into precision pest management (PPM) strategies for sustainable control of *P. canaliculata*.

## Simple Summary

The invasive freshwater snail *Pomacea canaliculata* severely jeopardizes agriculture, ecosystems and public health worldwide. The evolutionarily conserved azoospermia gene boule (bol) governs the metazoan spermatogenic process, but its function in molluscan species remains largely unknown. Pcbol was ubiquitously expressed across the life stages and tissues of *P. canaliculata*, notably in a male-biased manner. RNA interference (RNAi) substantially suppresses Pcbol at mRNA and protein levels, with underdeveloped reproductive glands and depleted sperm cell numbers, diminished soluble protein (SP)/testosterone (Te)/arginine (Arg) contents, and reduced egg-laying and hatchability. Mechanistically, CDC25 and other molecular modules were responsible for the fundamental role of Pcbol in male fertility. Our work highlighted Pcbol as a promising candidate for the RNAi-based male sterile technique (MST) to manage *P. canaliculata* populations.

## 1. Introduction

Gametogenesis, the generation of fully mature spermatozoa and oocytes, is a fundamental process in sexually reproducing animals [1]. The *Deleted in Azoospermia* (*DAZ*) gene family, consisting of highly conserved *boule*, *daz* and *dazl* (*DAZ-like*), is of paramount importance for meiosis, germ cell maintenance and gametogenesis across the animal kingdom [2,3,4]. Established as the progenitor of the *DAZ* family [5], *boule* was first identified in *Drosophila melanogaster* [6] and *Caenorhabditis elegans* (initially named *DAZ-1*) [7], and has since been well-documented as a critical modulator of male spermatogenesis in various metazoans [8,9,10,11,12], despite its occasional involvement in oogenesis [3,7,11,13]. While biological roles and evolutionary divergence of boule and its homologues have been extensively studied in both vertebrates and invertebrates, the molecular modules that operate downstream and/or in parallel with boule remain a black box.

Several lines of evidence signify the interplay between potential partners and boule. For instance, boule was responsible for initiating meiosis in male germline stem cells of dairy goats by directly activating stra8, scp3, cdc25a, cdc2 and vasa [14]. In addition, cdc25/twine (cdc25 orthologue) was translationally modulated by boule to direct G2/M stage meiosis in a testis-specific manner [6,15]. Similarly, Li et al. [12] underscored the critical role of *Sebol* in regulating male meiosis, particularly through interactions with syf1 and ZNFX1, which functioned in the G2/M transition. Boule was also implicated in the sperm differentiation pathway via tuning of modulo translation in *Drosophila* [16]. It was recently discovered that circular RNAs from boule coordinated with HSPs to rescue male fertility against heat stress in flies, mice and humans [17]. These interactions can be ascribed to the binding of the RRM domain and/or DAZ repeat of boule with response elements in the 3′-UTR of downstream effectors [18]. Whether other molecular components mediate the reproductive functions of boule warrants further exploration.

The golden apple snail (GAS), *Pomacea canaliculata* (Gastropoda: Ampullariidae) (Lamarck, 1822), is a freshwater mollusk endemic to South America [19]. Initially introduced into Guangdong province as an aquaculture food source in the early 1980s, the snail has since dispersed widely to 17 southern provinces in China [20], incurring massive economic losses per annum [21]. Because *P. canaliculata* poses grave threats to aquatic biodiversity, wetland ecosystems and services, agricultural crops and public health [22,23,24], it is notoriously recognized as one of the 100 worst invasive alien species (IAS) globally [25]. Due to its voracious appetite, rapid growth, high fecundity, robust adaptability to harsh environments and absence of natural enemies [26], biological, cultural and mechanical management of this plague mostly resulted in undesirable consequences [27]. Though this is not the case for chemical agents, the environmental pollution, nontarget risks and resistance development they induce cannot be dismissed [28]. It is therefore imperative to propose eco-friendly and effective strategies for GAS population control.

Originally conceptualized by Knipling [29], the male sterile technique (MST) has been developed as an eco-friendly alternative to synthetic chemicals and successfully applied to combat agricultural and sanitary pest populations, notably mosquitoes [30]. For MST implementation, infertile males are mass-produced by ionizing radiation or by genetic editing such as RNA interference (RNAi)-mediated gene silencing and/or knockout [31]. Due to slight mechanical injury and nil radiation-related risks, RNAi has emerged as a mainstay in the MST scheme, targeting key genes relevant to spermatogenesis or sperm viability [32], i.e., *Tssk1*, *Tektins*, *boule*, *double-sex male*, *Zpg*, *gas8*, *fzo*, etc. [12,33,34]. The R-strategy life history trait of *P. canaliculata,* featuring bisexualism and prolificacy, is the primary driver of aggressive population expansion in non-native ranges [35]. Recently, the comparative transcriptomic sequencing of *P. canaliculata* revealed male-specific genes *TSSK3/GnRHR2* and the involvement of *UBE2B*/*NDPK5* in spermatogenesis. Thus, MST targeting these genes holds great potential for suppressing GAS populations [36]. Likewise, given its biological significance for male fertility, *Pcbol* may be a promising candidate for RNAi-based MST [12].

With the aforementioned findings as the backdrop, we hypothesized that *Pcbol* may play a pivotal role in the male fertility of *P. canaliculata*. In this gene loss-of-function investigation, we depleted *Pcbol* through RNAi and integrated biochemical assays, qRT-PCR, Western blotting and immunofluorescence to (1) ascertain the involvement of *Pcbol* in physiochemical aspects of male reproduction and (2) unmask the molecular machinery that mediates *Pcbol* regulation in spermatogenesis. Here we presented the results of experiments testing the hypothesis.

## 2. Materials and Methods

### 2.1. Snail Husbandry and Collection

Adult individuals (7.0 ± 0.5 g) of uniform size were originally handpicked from a pond in Zhuanghang Town, Shanghai City, China (coordinates 30°53′29″ N, 121°23′16″ E). They were identified as *P. canaliculata* by morphological characterization and DNA barcoding of cytochrome c oxidase subunit I (COI) genes [37,38]. Kept in aquariums (80 cm long × 60 cm wide × 60 cm tall) containing dechlorinated water, snails were reared with fresh lettuce (*Lactuca sativa*) daily and acclimated to constant conditions of 24 ± 2 °C, 14 h light:12 h dark photoperiod and natural illumination. We adopted the method of Gamarra-Luques et al. [39] to distinguish sexes, where individuals bearing a concave operculum were determined to be females, and those with a convex shape toward the posterior margin were classified as males. Snails at different developmental stages were the progeny from the same batch of adults. During maintenance, female snails laid pinkish egg masses on the walls of the aquaria. A fraction of clutches were cautiously stripped off and harvested, while most others were left to hatch, fed following the same regime as adults and sacrificed after they grew into hatchlings (0.3 ± 0.1 g) and juveniles (1.5 ± 0.3 g). The tanks were covered with nylon nets, and tap water was purified using an overflow filter that was replenished weekly. Dead snails and food leftovers were removed daily.

### 2.2. Sequence Characterisation, Alignments and Phylogeny

We retrieved the genomic assembly, mRNA and protein sequences of Pcbol from NCBI GenBank databases (https://www.ncbi.nlm.nih.gov/, accessed on 15 August 2025). The Gene Structure Display Server (http://gsds.gao-lab.org/, accessed on 15 August 2025) was utilized to analyze the intron-exon organization. The ORF frame, conserved domains and repeated motifs were localized and visualized using https://www.ncbi.nlm.nih.gov/orffinder/, https://smart.embl.de/ and https://meme-suite.org/meme/tools/meme (all accessed on 15 August 2025). Boule orthologues from other genera were searched by the blastp program using >XP_025095407.1 as a query (https://blast.ncbi.nlm.nih.gov/Blast.cgi, accessed on 16 August 2025). Multiple sequence alignments were performed by Gene Doc v2.7.0 (http://nrbsc.org/gfx/genedoc/index.html, accessed on 16 August 2025) plus ClustalX v1.84 (http://www.clustal.org/clustal2/, accessed on 16 August 2025). The tertiary structure of Pcbol was deduced and constructed in SWISS-MODEL (https://swissmodel.expasy.org/interactive, accessed on 20 August 2025), and its quality was further evaluated using a Ramachandran plot and an ERRAT score. The cladogram of Pcbol and its homologues was established using the neighbor-joining (NJ) method in MEGA 7.0 with 100 bootstrap replicates (http://megasoftware.net/, accessed on 20 August 2025), and we used ChiPlot to beautify the phylogenetic tree (https://www.chiplot.online/, accessed on 20 August 2025). The physical and chemical parameters of Pcbol protein (Table S1) were computed using ExPASy ProtParam (https://web.expasy.org/protparam/, accessed on 18 March 2026).

### 2.3. RNA Isolation and dsRNA Synthesis

Specimens were homogenized using an SKXL homogenizer (BiHeng Biotechnology, Shanghai, China) at 4 °C, followed by RNA extraction with the SV Total Isolation System Kit plus genomic DNA (g DNA) Eraser (Promega, Madison, WI, USA). RNA yield and quality were assessed using a Nanodrop 2000 spectrophotometer (Thermo Fisher Scientific, Waltham, MA, USA), ensuring that the optical density ratio (OD A260/280) ranged from 1.8 to 2.0. RNA integrity was further validated by 1% formaldehyde agarose gel electrophoresis.

For dsRNA synthesis, a 205 bp fragment of *Pcbol* was initially amplified using primers that contain T7 RNA polymerase promoter sequences at the 5′ ends (Table S2). PCR amplification conditions comprised a denaturation at 94 °C for 2 min, followed by 35 cycles of 30 s at 94 °C, 30 s annealing at 56 °C, and 1 min extension at 72 °C, concluding with a final extension at 72 °C for 10 min. The resultant PCR product was gel-purified using a Gel Extraction Kit (Tiangen, Beijing, China), cloned into pMD18-T vector (Takara, Kyoto, Japan), and transformed into *Escherichia coli* DH5α competent cells (Vazyme, Nanjing, China). Positive clones were screened and validated by Sangon Biological Engineering Technology & Service Co., Ltd., Shanghai, China. The sequence-confirmed template was then utilized for in vitro transcription according to the manufacturer’s protocol for the T7 RiboMAX^TM^ Express RNAi System Kit (Promega, Madison, WI, USA). Sense and antisense single-stranded RNA (ssRNA) were synthesized independently in 20 µL reaction volumes, mixed, and annealed at 70 °C for 10 min, then cooled to ambient temperature over 20 min. To eliminate residual ssRNA and DNA, 2 µL RNase A solution (4 mg/mL) and 2 µL RNase-free DNase (1 U/µL) were introduced and then incubated at 37 °C for 30 min. The dsRNA was precipitated by adding 110 µL 95% ethanol and 4.4 µL 3 M sodium acetate (pH 5.2), washed with 0.5 mL 70% ethanol, air-dried, and dissolved in 50 µL nuclease-free water. As the negative control, lyophilized dsGFP (5 mg, 431 bp) was obtained from Shanghai Plant Science Biotechnology Co., Ltd. (Shanghai, China). Final concentrations of both dsGFP and dsPcbol were quantified via NanoDrop 2000 spectrophotometer (Thermo Fisher Scientific, Waltham, MA, USA) and diluted to a working concentration of 1 µg/µL.

### 2.4. cDNA Synthesis and qRT-PCR Analysis

Total RNA was initially isolated as outlined in Section 2.3. First-strand cDNA synthesis was performed using the PrimeScript^TM^ RT Reagent Kit with gDNA Eraser (Takara, Tokyo, Japan). Briefly, 1.0 µg RNA was mixed with 4 µL 5 × PrimeScript RT Master Mix and adjusted to 20 µL with nuclease-free ddH_2_O. Reverse transcription was carried out in a 9902 Applied Biosystems thermal cycler (Life Technologies, Foster, CA, USA) under the following conditions: 42 °C for 15 min, followed by 85 °C for 5 s. The yielded cDNA was diluted to 100 µL with ddH_2_O and stored at −20 °C until use.

qRT-PCR reactions were assembled in 20 µL volumes using the SYBR Color qPCR Master Mix (Vazyme, Nanjing, China), consisting of 10 µL SYBR Green mix, 0.5 µL each of forward and reverse primers (10 µM), 2 µL diluted cDNA template, and 7 µL ddH_2_O. Amplification was performed using a CFX96^TM^ Real-Time PCR Detection System (Bio-Rad, Hercules, CA, USA) with the following thermal profile: initial denaturation at 95 °C for 3 min, followed by 40 cycles of 95 °C for 10 s and 60 °C for 30 s. A melt curve analysis was conducted (60–95 °C, ramping at 0.5 °C per 5 s) to verify amplicon specificity and exclude primer dimers or genomic DNA contamination. Gene-specific primers were designed using the NCBI Primer-BLAST tool (http://www.ncbi.nlm.nih.gov/tools/primer-blast/, accessed on 20 October 2025) and were detailed in Table S2. GAPDH was employed as the endogenous reference gene for normalization [40]. Fold changes in gene expression were determined using the 2^−ΔΔCT^ method [41]. Each sample was loaded with two technical replications with three independent biological samples for each assay.

### 2.5. Experimental Design and Sample Collection

#### 2.5.1. Specimens for Expression Profiling

To perform qRT-PCR (Section 2.4) and blotting analysis (Section 2.7) for distinct developmental stages and tissues, samples of eggs/hatchlings/juveniles/females/males were harvested (two individuals or one egg mass per replicate, three replicates), with female ovary and male testis/pleopod/hepatopancreas/digestive gland/gill/mantle dissected and pooled (three samples per replicate, three replicates) for examination.

#### 2.5.2. dsRNA Delivery Protocol

As dsRNA concentrations and treatment durations affected RNAi silencing efficiency, delivery parameters were first screened and optimized based on three doses (2, 4, and 8 µg/snail) at four time points [42]. Prior to injection, snails were immobilized by placing them on ice for 15–20 min. Once anesthetized, the operculum was carefully retracted, and dsRNA solution was administered into the pleopod muscle using a sterile Hamilton 701 RN microsyringe (Hamilton, Whittier, CA, USA). The needle was maintained in position for 10 s post-injection to avoid solution leakage. *Pcbol* transcript levels (three testis tissues/group) were measured using qRT-PCR (Section 2.4) at 1/3/5/7 days post-injection, and the mortality was recorded (50 males/group). The optimal delivery parameters were determined according to desirable knockdown efficacy and acceptable survival rates. Our previous work [42] confirmed that the dsGFP negative control induced no significant alterations in the expression levels of target genes. Therefore, a control injection was implemented with an equivalent volume of dsGFP relative to the dsPcbol treatment, with three replicates per group.

#### 2.5.3. Sampling of RNAi-Males for Functional Characterization

Taking the death toll post dsRNA administration into account, at least 50 Pcbol-RNAi males and 35 dsGFP-treated males were recruited. Specifically, for biochemical tests, three testes were pooled as one sample, with three samples per group (*n* = 3). For the fertility bioassay, ten mating parallels were set for each group (*n* = 10), with one male and female couple in each parallel. For sperm count and morphology examination, three testes were dissected from each treatment (*n* = 3), and each tissue was aligned with one observation. For qRT-PCR analysis of spermatogenic genes, three testes were sacrificed as one sample, and each treatment consisted of three samples (*n* = 3). For Western blotting and immunofluorescence assays, each assay comprised six testes and three for each group (*n* = 3), with one testis corresponding to one measurement.

### 2.6. Western Blotting

Testes tissues were lysed using the Column Tissue & Cell Protein Extraction Kit (Epizyme Biotech, Shanghai, China), and total protein concentrations were determined via the bicinchoninic acid (BCA) assay (CWBIO, Beijing, China). Equal amounts of protein (30 µg) were combined with 6× loading buffer (Beyotime, Shanghai, China) and denatured by boiling for 10 min. After centrifugation at 12,000× *g* for 10 min and cooling to room temperature, samples were subjected to SDS-PAGE using a 4% stacking gel and 12% resolving gel in a Mini-Protean electrophoresis apparatus (Bio-Rad, Hercules, CA, USA) at 120 V for 100 min. Resolved proteins were electrotransferred onto 0.45 µm nitrocellulose membranes (Beyotime, Shanghai, China), which were subsequently rinsed with Tris-buffered saline (TBS) for 5 min. Membranes were blocked for 1 h at room temperature in TBS supplemented with 0.1% Tween 20 (TBST) and 5% (*w*/*v*) skim milk, followed by three washes of 5 min each in TBST. Blots were then incubated overnight at 4 °C with primary antibodies diluted 1:2000 in TBST, washed three times for 10 min each in TBST, and subsequently probed with HRP-conjugated goat anti-rabbit IgG (Beyotime, Shanghai, China) diluted 1:3000 in TBST. Following extensive washing, immunoreactive bands were visualized using an enhanced chemoluminescence (ECL) detection kit (Beyotime, Shanghai, China) in the Molecular Imager ChemiDoc XRS System (Bio-Rad, Hercules, CA, USA). Band intensities were quantified by densitometric analysis using ImageJ software (version 1.53, Wayne Rasband, MD, USA). Primary antibodies employed included a custom-synthesized Pcbol rabbit monoclonal antibody (GenScript Biotech Corporation, Nanjing, China) and a GAPDH rabbit monoclonal antibody (Beyotime, Shanghai, China), the latter serving as the internal loading control.

### 2.7. Immunofluorescence Microscopy

To detect the distribution and localization of Pcbol upon dsRNA injection, immunofluorescence was performed as described by Wu et al. [32]. Testes dissected from male GAS were fixed in 4% paraformaldehyde (in PBS, *w*/*v*) for 2 h at 4 °C, rinsed with PBS for 3 × 10 min (0.01 M, pH 7.2–7.4), then permeabilized in PBS plus 0.2% (*w*/*v*) Triton X-100 for 30 min prior to being blocked by 3% (*w*/*v*) BSA for 4 h at room temperature. After washing in PBS (3 × 5 min), the tissues were incubated with Pcbol primary antibody for 24 h at 4 °C, diluted 1000-fold in PBST containing 3% BSA. This monoclonal antibody was raised from rabbit serum against the RRM domain (31–97 aa), synthesized and purified by GenScript Biotech Corporation (Nanjing, China). In darkness, samples were eventually probed using Alexa-594-tagged goat anti-rabbit secondary antibody (1:2000, Thermo Fisher Scientific, Waltham, MA, USA) at 4 °C for 2 h and counterstained with 1 μg/mL DAPI (100 nM, Beyotime, Shanghai, China) for 10 min at room temperature. Post PBS rinsing (3 × 5 min), we mounted specimens on glass slides and observed them under an LSM 780 confocal microscope (Zeiss, Jena, Germany). The excitation wavelengths for DAPI (blue) and Pcbol (red) were captured at 405 and 594 nm, respectively, with images photographed using a Zeiss AxioCam M5Rc digital camera (Zeiss, Jena, Germany).

### 2.8. Biochemical Measurements and Fertility Bioassay

Levels of soluble protein (SP), testosterone (Te), and arginine (Arg) in testes taken from males subjected to Pcbol knockdown were determined using a total protein assay kit (Nanjing Jiancheng Bioengineering Institute, Nanjing, China), a testosterone assay kit (Nanjing Jiancheng Bioengineering Institute, Nanjing, China) and an arginine ELISA Kit (AVIVA Systems Biology, Beijing, China) according to specifications. In brief, samples were immersed in 100 mM cold phosphate-buffered saline (PBS, pH = 7.2–7.4) (1:9, *w*/*v*), homogenized using the SKXL homogenizer (Biheng Biotechnology, Shanghai, China) and then centrifuged at 12,000× *g* for 30 min at 4 °C (Eppendorf centrifuge 5418R, Hamburg, Germany) to yield supernatants for bioassays. Absorbance at the wavelengths of 595 nm, 240 nm and 450 nm was registered in a UV-2550 spectrophotometer (Shimadzu, Tokyo, Japan). Each treatment and control was performed thrice with three independent biological samples.

Male fertility was examined according to previously reported methods, with modifications [43]. Wild *P. canaliculata* populations were identified, sampled and reared as described in Section 2.1. Egg masses laid by the same batch of wt females were left to hatch under controlled indoor conditions. The hatchlings were incubated for two months, followed by separation rearing of male and female individuals for another month. Afterwards, adult males were treated with dsPcbol and dsGFP, respectively, and paired with wt females at a 1:1 ratio. Post 72-h copulation, wt females were isolated and fed individually, with their total egg production recorded over the next 15 days [44]. All deposited egg clutches were recruited, transferred to an open breeding chamber (42 cm × 20 cm × 26 cm, with tap water to maintain humidity) [45] and monitored for an additional 15 days to register hatching rate. Notably, the remaining non-hatched eggs within egg masses were tallied after they were dispersed in 2% sodium hydroxide solution for 1.5 h. Each group consisted of 10 mating pairs, with dsGFP as the parallel control.

### 2.9. Phenotypic Analysis of Sperm Cell and Reproductive Gland

The total number of sperm cells was determined according to the protocol of Marciniak et al. [46]. Following dsRNA-mediated silencing, testes were dissected and washed in PBS (0.01 M, pH 7.2–7.4). Testes were opened via fine forceps, and then the vas deferens was amputated by surgical blades to release and harvest semen. Subsequently, a suspension of spermatozoa in PBS was vortexed, air-dried for 30 min and fixed in a solution of 4% paraformaldehyde for 15 min. Fixed cells were stained for 15 min with DAPI (100 nM, Beyotime, Shanghai, China) and were then rinsed and mounted on glass slides with mounting medium (90% glycerol, 2.5% DABCO, PBS). Samples were examined using a DM 5000B microscope coupled to a DFC300FX camera (Leica Microsystems, Wetzlar, Germany). The reproductive glands were anatomized from adult males of each group. Afterwards, the morphology was photographed under the dark field using a Nikon SMZ18 stereomicroscope equipped with a DS-Fi2 camera (Nikon, Tokyo, Japan). The length and width of reproductive glands, as well as the number of spermatogenic cells, were averaged from three independent repetitions, each measured by ImageJ software (version 1.53, Wayne Rasband, MD, USA).

### 2.10. Homology Modelling and Molecular Docking

Due to the absence of available PDB structures for Pcbol and CDC25 in the RCSB PDB databank, the three-dimensional (3D) structures of these proteins were predicted from their respective amino acid sequences using SWISS-MODEL (https://swissmodel.expasy.org/, accessed on 10 December 2025). The dominant conformation with the highest score was considered the definitive structural model for analysis. Structural preprocessing was subsequently operated in MGTools v1.5.6 (https://ccsb.scripps.edu/mgltools/1-5-6/, accessed on 10 December 2025), encompassing water molecule elimination, hydrogen addition, charge assignment, and non-polar hydrogen merging. Afterwards, the refined models were amenable to docking simulations in the ZDOCK program (https://zdock.umassmed.edu/, accessed on 10 December 2025), and each binding model was evaluated using an energy-based scoring function. Among 1000 docking trials, the docking conformation with the lowest binding energy was considered the optimal Pcbol-CDC25 complex. Molecular docking and interactions were visualized using PyMOL v.2.5.2 (DeLano Scientific, San Carlos, CA, USA), and the binding free energies (ΔGbind) were analyzed using PDBePISA (https://www.ebi.ac.uk/msd-srv/prot_int/cgi-bin/piserver, accessed on 10 December 2025).

### 2.11. Statistics

All analyses were performed in the Data Processing System (DPS) software (version 7.05, Hangzhou, China) [47]. Data are presented as mean ± SEM (standard error of mean) from at least three independent biological replicates unless otherwise stated. Comparisons between two samples were evaluated by unpaired two-tailed Student’s *t*-tests and one-way analysis of variance (ANOVA), followed by Tukey’s post-hoc test, which was applied for multiple treatments, with the statistical significance versus control set as *p* < 0.05 (single asterisk) or *p* < 0.01 (double asterisk). All graphs were visualized using GraphPad Prism (version 9.0.0, San Diego, CA, USA), where significant differences between survival curves were assessed by the Log-rank (Mantel-Cox) test (** *p* < 0.01).

## 3. Results

### 3.1. Sequence and Structure Analysis of Pcbol

**Figure 1:**
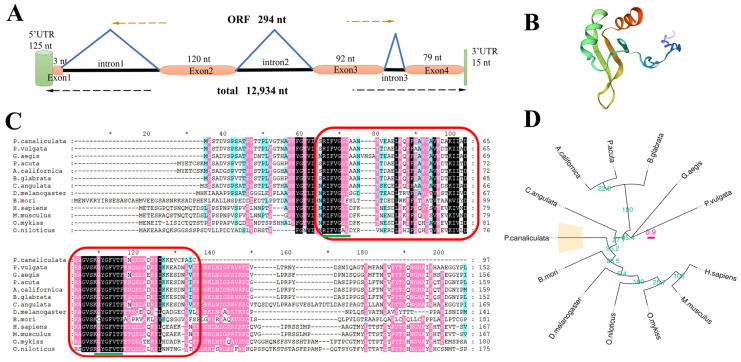
Molecular characterisation of Pcbol cDNA and protein sequences. (**A**) Schematic illustration of Pcbol genomic organisation. (**B**) The deduced tertiary structure of the Pcbol protein. (**C**) Multiple alignments of boule amino acid sequences among different species. Red frames denote the RNA recognition motif superfamily (31–97 aa), where conserved ribonucleoprotein domains including RNP-2 motif (RIFVGGI) plus RNP-1 motif (KGYGFV/ITF) are underlined by green bars. Residues shaded in black, pink and cyan are identical (100%), conserved (80%) or similar (60%) in terms of homology. The asterisk (*) denotes an interval distance of 10 aa. GenBank accession numbers and species abbreviations: XP_025095407.1 [*Pomacea canaliculata*], XP_055959301.1 [*Patella vulgata*], XP_052682382.1 [*Crassostrea angulata*], KAI8793261.1 [*Biomphalaria glabrata*], XP_059152941.1 [*Physella acuta*], XP_041365107.1 [*Gigantopelta aegis*], XP_005103136.2 [*Aplysia californica*], NP_001106740.1 [*Bombyx mori*], NP_932074.1 [*Homo sapiens*], NP_729457.1 [*Drosophila melanogaster*], XP_021426586.2 [*Oncorhynchus mykiss*], XP_003455956.1 [*Oreochromis niloticus*], NP_083543.2 [*Mus musculus*]. (**D**) Phylogenetic relationships between Pcbol and orthologues from other organisms. Numbers at branch nodes represent bootstrap values (1000 replicates). Scale bar indicates the number of amino acid substitutions per site. The protein boule-like isoform 1 [Homo sapiens] is chosen as the outgroup.

The full-length genomic DNA of *Pcbol* was 12,934 nt, comprising a 125-nt 5′ untranslated region (UTR), 15-nt 3′UTR and an ORF of 294-nt encoding 97 aa residues (Figure 1A). This protein sequence shared the maximal (91.21%) and lowest (54.41%) similarity with homologs from *Patella vulgate* and *Oreochromis niloticus*, respectively. Highly conserved positions among boule orthologues resided within the RRM region, where two emblematic motifs, RNP-2 and RNP-1, were illustrated (Figure 1C). A total of two helices, three strands and six coils were assembled in the Pcbol tertiary structure (Figure 1B). As depicted in the dendrogram, the Pcbol protein clustered with its *Bombyx mori* counterpart to form one clade. It also presented an intimate phylogenetic relation with orthologues of *Crassostrea angulata* and other molluscan species (Figure 1D). The physicochemical properties of Pcbol protein were detailed in Table S1.

### 3.2. Spatiotemporal Expression Profiling of Pcbol

**Figure 2:**
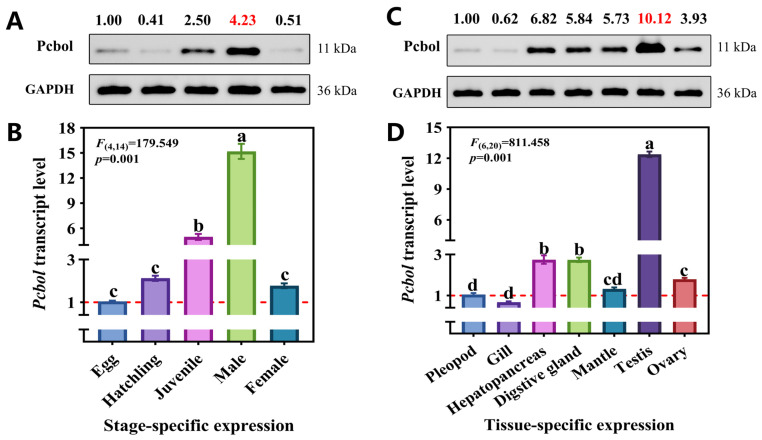
The spatial–temporal expression profiles of Pcbol. (**A**,**B**) Relative protein and mRNA levels of Pcbol at different development stages. (**C**,**D**) Relative protein and mRNA levels of Pcbol in various male tissues and female ovaries. Relative expression levels are normalized to GAPDH with values of egg and pleopod as calibrators. Each histogram and error bar represents mean ± SEM (*n* = 3). Different lowercase letters denote significant differences among groups (*p* < 0.05, one-way ANOVA followed by post-hoc Tukey’s test). Relative protein levels are listed above corresponding bands and calculated as a ratio of grey values, viz., target protein divided by GAPDH.

As shown in Figure 2B,D, *Pcbol* was universally transcribed across all developmental stages and adult tissues, specifically with preferential transcriptions for juvenile and male snails as well as male digestive glands and testes. Due to the circa 6.9- and 8.5-fold higher abundance in males/male testes than females/female ovaries, respectively, it was noticeable that *Pcbol* was enriched in a male-biased manner. Albeit discrepancy in fold changes, the expression trend was concurrent at the protein level (Figure 2A,C and Figure S1 (middle, right)), as Pcbol peaked at male stage and male testis tissue, except for slight mitigation at hatchling stage, which may be attributed to posttranscriptional and posttranslational modifications, and differential degradation rates between mRNA and protein in a specific tissue and at a specific stage [48].

### 3.3. Pcbol Silencing Induced by dsRNA Injection

To ensure RNAi efficiency and produce sufficient individuals for sample collection, we set up a gradient concentration of dsRNA alongside multiple treatment durations. Adult males receiving dsPcbol injections exhibited dose-dependent lethality of 4.0–46.0% across 2–8 μg at 1/3/5/7 days (Figure S2A). In parallel, dsGFP treatments rendered only one or two individuals dead, validating its suitability as the negative control. qRT-PCR analysis displayed that administration of 4 µg dsPcbol for 1, 3, 5, and 7 days achieved 32.2–70.3% knockdown (Figure S2B), which peaked at the 5th day post-treatment and was comparable to silencing efficacy induced by the highest dose (8 µg), but the mortality was substantially lower. By assessment of both knockdown efficacy and snail mortality, 4-µg dsPcbol delivery and day 5 sampling post-injection were determined as optimal parameters.

**Figure 3:**
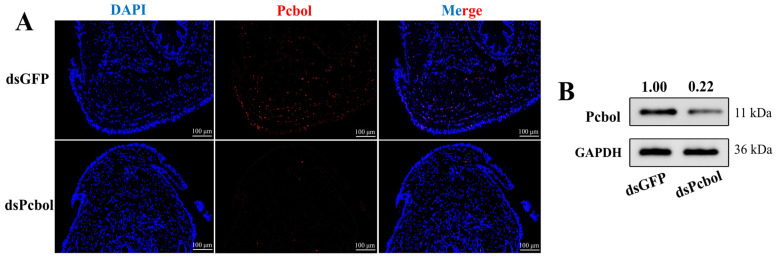
Translation levels of Pcbol in male testes upon dsRNA injections. (**A**) The localization and expression are illustrated by fluorescent intensity. Spermatozoan nuclei and Pcbol antibody are labelled by DAPI (blue) and Alexa-594 (red), respectively. Scale bar, 100 μm. (**B**) Western blotting with GADPH as the loading control. Relative grey values are normalized to GADPH and marked above corresponding bands, where 1.00 is set for the dsGFP group.

RNAi assays were performed based on the above conditions. Immunofluorescence analysis of testes from dsPcol-injected males clearly manifested a faint signal of Pcbol, while heightened intensity was evenly distributed in the dsGFP group (Figure 3A). As expected, Pcbol protein level also experienced a pronounced decrease following Pcbol silencing, down by circa 78.0% relative to the dsGFP (Figure 3B and Figure S1 (left)).

### 3.4. Pcbol Depletion Impaired Male Fertility

**Figure 4:**
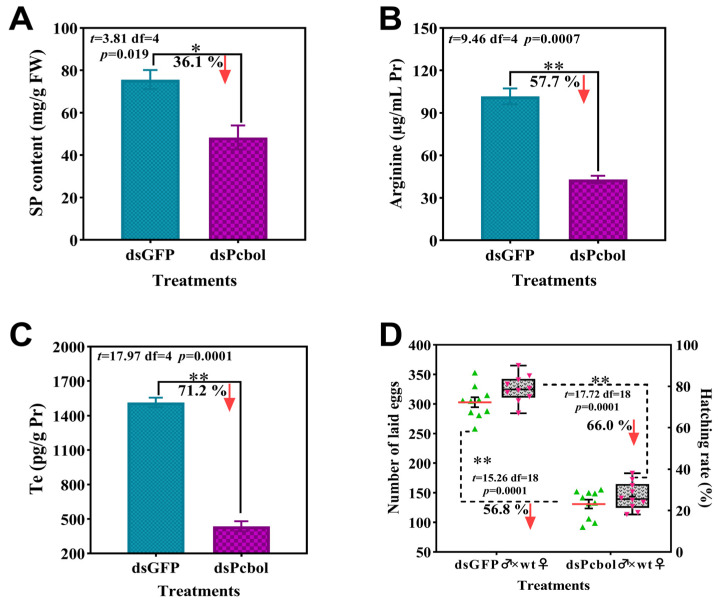
Effects of *Pcbol*-RNAi on biochemical indexes correlated with male fertility. (**A**) SP content (mg/g fresh weight) (*n* = 3); (**B**) Arginine (µg/mL protein) (*n* = 3); (**C**) Te (pg/g protein) (*n* = 3); (**D**) Number of laid eggs by wild type females and egg hatching rate (%) (*n* = 10). Each green/red triangle, red/black horizontal line denotes raw data and median values, with “+” indicative of means. Boundaries of the box plot signify 25/75th percentiles, and whiskers indicate maximum and minimum. Each histogram and error bar represents the mean ± SEM. Statistical comparisons are performed using Student’s *t*-test, and asterisks denote significant differences (* *p* < 0.05, ** *p* < 0.01).

Relative to the dsGFP group, testes of males subjected to dsPcbol injection harbored significantly diminished levels of SP, Arg and Te, down by 36.1%, 57.7% and 71.2%, respectively (Figure 4A–C). Furthermore, after Pcbol-RNAi male copulation with wild-type female counterparts, proxies like the number of deposited eggs per female and the ratio of incubated eggs were all significantly suppressed by 56.8% and 66.0% relative to the dsGFP♂ × wild-type ♀ group (Figure 4D), respectively.

### 3.5. Pcbol-RNAi Disrupted Sperm Production and Arrested Reproductive Gland

**Figure 5:**
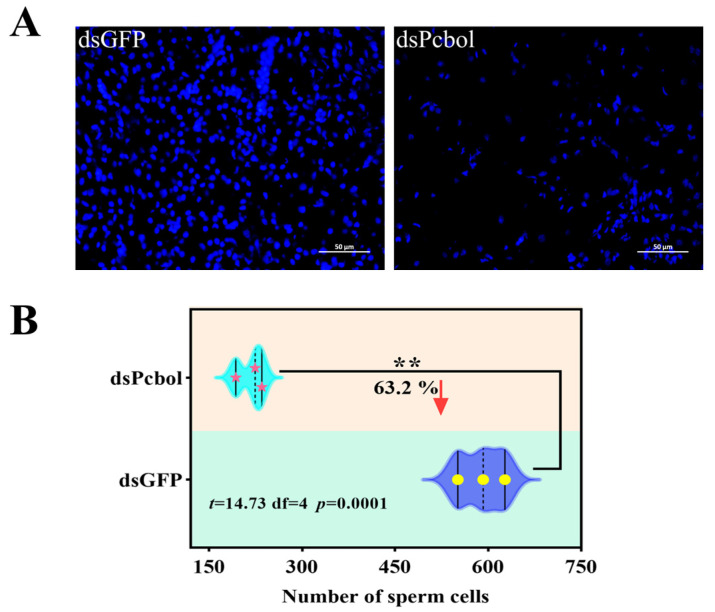
Representative micrographs (**A**) and relative number (**B**) of spermatozoan nuclei from males subjected to *Pcbol* silencing. The observations and calculations are performed thrice, with a dashed line and a symbol denoting the median value (Student’s *t*-test, ** *p* < 0.01). Scale bar, 50 μm.

**Figure 6:**
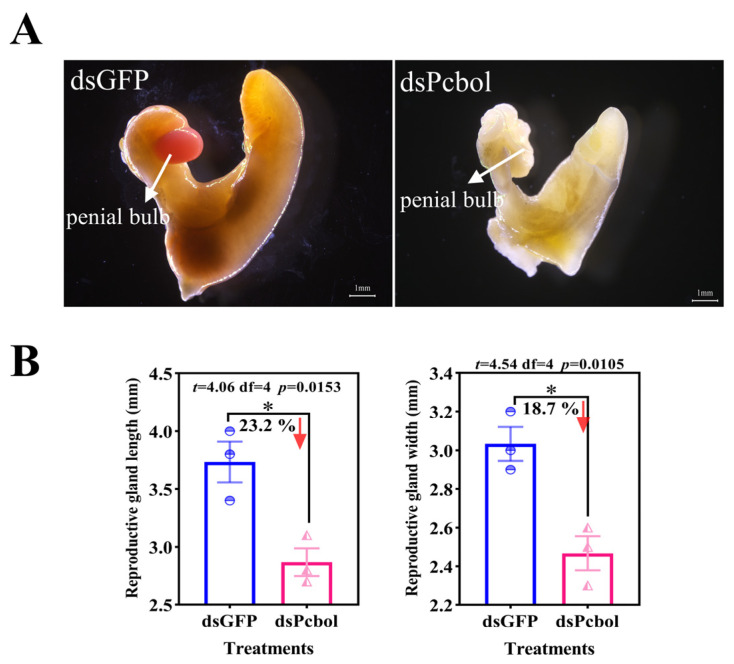
Anatomical pictures of the reproductive gland (**A**) and size measurement (**B**) post *Pcbol* depletion. The penis bulb is highlighted by the white arrow. Each histogram and error bar represents mean ± SEM (*n* = 3), with different symbols denoting a specific value and an asterisk indicative of a significant difference (Student’s *t*-test, * *p* < 0.05). Scale bar, 1 mm.

Opposite to the densely packed nuclei with sharp outline and intact morphology in the dsGFP group, indistinct outline, widened intercellular spaces and decreased cellular density were observed for sporadic spermatozoan nuclei following *Pcbol* knockdown. Besides, circa 63.2% diminished cell quantities reflected that a spermatogenic failure occurred in dsPcbol-treated males (Figure 5). RNAi-targeting *Pcbol* rendered visible degeneration of the penis bulb and stunted phenotype of the reproductive gland (Figure 6), with significant reductions of 23.2% and 18.7% in both length and width, respectively.

### 3.6. Effects of dsPcbol Treatment on Molecular Markers of Spermatogenesis

**Figure 7:**
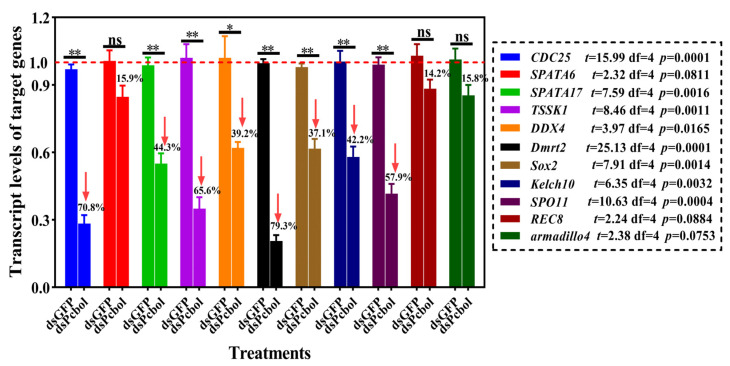
Effects of *Pcbol* knockdown on expression profiling of spermatogenesis-related genes. Transcript level of each target gene is normalized to the internal reference *GAPDH*. Each histogram and error bar represents mean ± SEM (*n* = 3 per group). Statistically significant differences are followed by asterisks (Student’s *t*-test, * *p* < 0.05, ** *p* < 0.01), ns, not significant.

In terms of spermatogenesis-relevant genes (Table S2), *Pcbol* depletion led to differential downregulation of all tested targets, significantly reducing by 37.1–79.3% relative to the dsGFP group (Figure 7), with the highest suppression for *Dmrt2*, *CDC25* and *TSSK1*. Conspicuous yet not significant diminution was witnessed for REC8, *SPATA6* and *armadillo4*.

**Figure 8:**
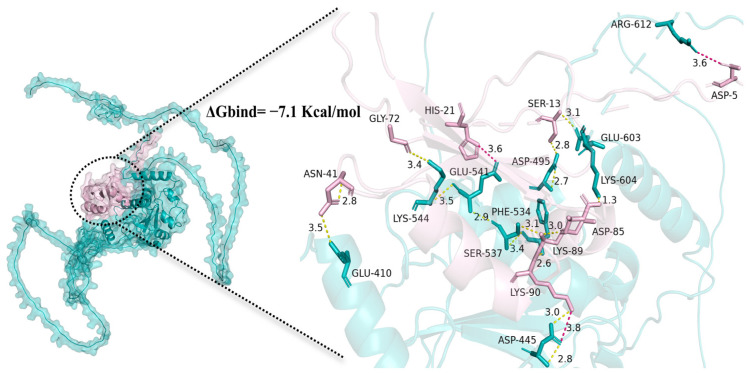
In-silico docking model based on protein structures of Pcbol (pink) and CDC25 (cyan). The peptide binding interface was circled and enlarged in the right panel, where hydrogen bonds (yellow) and salt bridges (red) were labelled by dashed lines. Distances (Å) of residue interactions were presented numerically.

The docking analysis revealed strong affinity and intermolecular forces between the Pcbol-CDC25 complex (Figure 8), with a binding capacity (ΔG) of −7.1 Kcal/mol and five amino acid residues forming hydrogen bonds in the RRM domain of Pcbol, i.e., ASN-41, GLY-72, LYS-89, ASP-85 and LYS-90.

## 4. Discussion

Gonochorism and gametogenesis constitute cornerstone physiology that subserves copulation, reproduction and population proliferation across animal phyla. This is especially paramount for oviparous *P. canaliculata*, whose invasive success depends on aggressive interspecific competition within shared ecological niches. Whereas most studies have dealt exclusively with female reproductive investment but overlooked contributions of parental males [49]. Here we focused on a male fertility factor, boule, which falls into the conserved DAZ family responsible for development, differentiation and maintenance of male germline cells and reproductive capacity [6,8,9,10,11,12,50,51,52]. To the best of our knowledge, this is the first report unravelling the biological functions of boule in a molluscan model.

Generally, alternatively spliced isoforms of *boule* exhibited unisexual expressions that considerably varied among invertebrates and vertebrates [1,13,53]. For instance, *boule* was uniquely transcribed in male testes of fruit flies and mammals [5,6,9,54]; as to nematode homologues, *boule* mutation completely aborted female oocyte production but not male spermatogenic progression [7]. This phenomenon is similar in flatworms, with ovary-biased expression of *macbol3* exclusively essential for oogenesis [3]. By contrast, the presence of *boule* paralogs in both male and female gonads was documented in diverse fish [2,11,13,53] and insect species [1,12,51,55]. Although the distribution of Pcbol was constitutively detected in the ovary and somatic tissues, the testis-specific and male-biased accumulation was noticeable (Figure 2 and Figure S1), suggesting it may be implicated in male fertility modulation. However, our study only identified one boule gene homologue in the *P. canaliculata* genome and whether additional isoforms exist with functional divergence merits further exploration.

Outcomes of this study strongly lent support to the previous argument [11,55] and our assumption that boule served as a core genetic marker for spermatogenesis and male fertility. Typical azoospermic phenotype and fertility suppression were observed upon *Pcbol* knockdown, as evidenced by substantial deficiency of sperm production (Figure 5), SP/Arg/Te contents alongside eggs laid and hatchability (Figure 4). These findings resembled investigations of sawfly [51] and beet armyworm [12], where lack of mature sperm and male sterility were registered due to boule loss-of-function. This was also true for the asexually-reproduced *Macrostomum lignan*, with the testis devoid of mature spermatozoa after either *macbol1* or and *macbol3* depletion and RNAi-treated flatworms fathered fewer offspring than the control [3]. SP and Arg are not only essential nutrients for the protection and nourishment of spermatozoa, but also can be utilized as nuptial gifts transferred from males to females during copulation, facilitating the fertilization chance and reproductive success [56,57,58,59]. Hence, diminished amino acid and protein levels severely compromised sperm quality (motility, function, etc.) and acrosomal membrane structure [59,60,61,62], finally triggering fewer eggs to be fertilized and hatched. Testosterone is the primary male steroid hormone endocrinologically synthesized in the testis [63]. Aligned with the suppressed sperm quantity in our study, disruption of the boule gene or testosterone signaling also led to spermatogenic arrest in rainbow trout [64]. Apart from the nutritional and endocrine aspects, it should be noted that aberrant and impeded MRG appeared in dsPcol treatment (Figure 6), signifying the pivotal role of *Pcbol* in male gonad development. Overall, our results suggested that Pcbol may modulate male reproductive physiology by directly affecting MRG development or indirectly perturbing testis nutritional status and hormonal homeostasis.

Basically, spermatogenesis is fine-tuned by complicated interactions among multiple genes and signaling pathways. Any deviation from the normal expression of any one or more of these genes may lead to spermatogenic arrest [52,65]. Herein, transcripts of all spermatogenic genes dwindled in *Pcbol*-RNAi males, and we identified *Dmrt2*, *CDC25* and *TSSK1* as the three most sensitive genes prone to *Pcbol* silencing (Figure 7, Table S2), of which the *Dmrt* and *TSSK* gene families were confirmed as markers crucial for molluscan spermatogenesis and fertility [36,66]. Therefore, *Pcbol* may exert functions by the orchestration of these key genetic modules. Reports by Dong et al. [31] align with our finding that the disruption of spermatogenesis-related genes mitigated the number of spermatozoa in the female spermatheca, eventually resulting in reduced fertility. Intriguingly, *Pcbol* depletion significantly suppressed *SPATA17* transcript levels but not *SPATA6*, suggesting overlapping roles and/or compensatory actions of alternative spliced variants within the same gene family.

Structurally, boule homologues feature a conserved RNP-type RRM domain for binding target mRNAs [1,3,8,67]. Similarly, the Pcbol amino acid sequence shared high identity and close phylogenetic relationship with counterparts from insects, mollusks, and mammals (Figure 1). This is echoed by the fact that boule orthologues are highly conserved among metazoans with little intra-specific variation, which implies their general property of transcription or translational control and highly-selected, essential function [51,68]. Given the unique binding property and translational regulation of CDC25 by Pcbol [4,15,50,51], we further performed molecular docking to model their interactions. As anticipated, robust affinity and intermolecular forces were formed and lay in the RRM domain, corroborating the Pcbol-CDC25 regulatory module at both transcriptional (Figure 7) and silico level (Figure 8). Additionally, as the meiotic progression indicator, CDC25-RNAi also caused the absence of mature sperm and mature eggs in sawfly [51], mimicking the phenotype induced by boule mutation. Thus, whether Pcbol may act independently of or synergistically with CDC25 to regulate male reproductive fitness remains to be answered.

Still, the eradication of GAS infestation routinely involves the use of molluscicides, which present nontarget hazards and environmental concerns. MST constitutes an alternative to chemical agents by manipulating male-fertility related genes based on gene editing approaches like RNAi, a reverse genetic method to post-transcriptionally silence endogenous mRNAs [31,33]. As the next-generation pest management strategy, RNAi also holds great promise to effectively control invasive species [1,69]. This study fulfilled the robust and persistent RNAi effect in that dsRNA administered over 4 µg considerably depleted Pcbol expressions by 32.2–78.0% (Figure S2 and Figure 3B), which can last for 7 days post-delivery (Figure S2), indicating a systematic RNAi response [70]. Furthermore, the high silencing efficacy was comparable to our previous finding [42] and other gene loss-of-function studies regarding *P. canaliculata* [67,71], further reinforcing the practical viability and prospective application of RNAi in this mollusk. Our investigation pointed to Pcbol as a central player in spermatogenesis and a critical marker of male fertility. In light of MST and PPM notions, boule can be harnessed to develop an RNAi-based sterilant to combat GAS population growth.

## 5. Conclusions

In summary, this is the first report focusing on the biological roles of *boule* in a molluscan model from a male perspective. The genomic organization, protein structure, amino acid sequences and phylogenetic relations of Pcbol were characterized, with its mRNA and protein preferentially expressed in adult males, notably testes. As reflected by suppressed expressions of Pcbol both transcriptionally and translationally, dsPcbol induced effective systemic RNAi in *P. canaliculata*. Upon *Pcbol* knockdown, the reduced levels of Te/Arg/SP, retarded reproductive gland, accompanied by a mitigated number of germline cells, laid eggs and egg hatchability all pointed to fundamental significance of Pcbol in male fertility, which may be mediated by interactive partners (CDC25, etc.) pertaining to spermatogenic meiosis and sperm development. These results may aid in the RNAi-based MST to manage invasive populations of this pest.

## Data Availability

Data are contained within the article or Supplementary File.

## Supplementary Materials

The following supporting information can be downloaded at: https://www.mdpi.com/article/10.3390/biology15070554/s1, Table S1. Physicochemical properties of Pcbol protein. Table S2. Oligonucleotide primer sequences for qRT-PCR assays and dsRNA synthesis. Figure S1. Original Western blotting images for Figure 3B (left), Figure 2A (middle) and Figure 2C (right). Figure S2. The survival rate of individual male (A) and *Pcbol* expressions of testes (B) subjected to different concentrations (2/4/6/8 μg) of dsRNA during multiple time periods (1/3/5/7 day). Significant differences in survival are assessed by logrank (Mantel-Cox) test in GraphPad Prism (*n* = 50, *p* < 0.0001). The mRNA levels of *Pcbol* are compared using one-way ANOVA followed by post-hoc Tukey’s test, with asterisks indicative of significant differences relative to the control group (dsGFP) at the same concentration and time point (** *p* < 0.01). Data are presented as mean ± SEM (*n* = 3). References [72,73,74,75,76,77,78,79,80] are cited in the Supplementary Materials.

## Abbreviations

heat shock proteins (HSPs), cell division cycle 25 phosphatase (cdc25), stimulated by retinoic acid gene 8 (stra8), Deleted in Azoospermia (DAZ), meiosis (reductional maturation divisions), 3′-UTR (untranslated regions), RRM (RNA recognition motif), synaptonemal complex protein 3 (scp3), testosterone (Te), phosphate-buffered saline (PBS), bovine serum albumin (BSA), 4′,6-diamidino-2-phenylindole (DAPI), RNA interference (RNAi), Testosterone (Te), Soluble protein (SP), nucleotide (nt), amino acid (aa), base pair (bp), Quantitative real-time PCR (qRT-PCR), probability value (*p*), green fluorescence protein (GFP), double-stranded RNA (dsRNA), sodium dodecyl sulfate polyacrylamide gel electrophoresis (SDS-PAGE), male sterile technique (MST), PBST (phosphate-buffered saline with 0.1% Tween 20), precision pest management (PPM), wild type (wt), RNP (ribonucleoprotein), MRG (male reproductive gland).
