# Supplementary material for: The Molecular Characterization and Functional Analysis of Pomacea canaliculata Boule: A Central Player in Spermatogenesis and Male Fertility
Source: Biology (Basel). 2026 Mar 30;15(7):554. doi: 10.3390/biology15070554 (PMC13072017; doi:10.3390/biology15070554)
Supplement: Supplementary file 1 [file biology-15-00554-s001.zip › biology-4180093-supplementary.pdf]

Table S1. Physicochemical properties of Pcbol protein.

Isoelectric point	Molecular weight	Number of residues	Predicted locations	Instability index	Aliphatic index	Grand average of hydropathicity (GRAVY)
6.55	10,228.68 g/mol (10.23 kDa)	97 aa	Cytoplasm	22.12 (Stable)	79.48	0.153 (Hydrophobicity)

Table S2. Oligonucleotide primer sequences for qRT-PCR assays and dsRNA synthesis.

Gene name	GenBank Accession No.	Sequences (5′–3′)	Purposes	Amplicon length (bp)	Functions & references
glyceraldehyde-3-phosphate dehydrogenase-like (*GAPDH*)	XM_025226527.1	Forward: CAACCTCAAAACCGATGCCA<br>Reverse: GACAAAGCGATTAGTCAGTGGA	qRT-PCR	184	housekeeping gene for normalization [40]
cdc25-like protein phosphatase twine	XM_025240861.1<br>XM_025240862.1	Forward: ATCCGCAGCAAGAGTTAGGC<br>Reverse: CTGAGATTCATCGCCAGGCT	qRT-PCR	145	downstream target of boule, crucial for G2/M transition

(*CDC25*)					of meiosis [9,51,72]
ATP-dependent RNA helicase DDX4 (*DDX4/vasa*)	XM_025221783.1	Forward: CCAAGCCTACCCCAATCCAG<br>Reverse: CCAGTCTGAGCACAACCCAT	qRT-PCR	79	essential for male germ cells to progress through spermatogenesis [73]
testis-specific serine/threonine-protein kinase 1-like (*TSSK1*)	XM_025249072.1	Forward: GCCCTACGACGATTCCAACA<br>Reverse: GTCCTTGGCCTCGTTTGAGA	qRT-PCR	100	testis-specific gene essential for spermatogenesis, male fertility and differentiation of spermatozoa [36,66,74]
spermatogenesis-associated protein 17-like (*SPATA17*)	XM_025249196.1	Forward: CTCCTTTCCTTCCGTGTCCC<br>Reverse: GGTTCTCGGAATGGACCCTG	qRT-PCR	181	responsible for gonadogenesis and testis development [75]
spermatogenesis-associated protein 6-like (*SPATA6*)	XM_025253584.1<br>XM_025253585.1<br>XM_025253586.1<br>XM_025253587.1<br>XM_025253588.1	Forward: CTGGCATTTCCCCCAAGCTA<br>Reverse: GTCTTGTAGGGCTTCGCCTT	qRT-PCR	144	a critical protein in the assembly and/or structural integrity of the sperm tail axoneme [76]

Kelch-like protein homolog 10 (*Kelch10*)	XM_025240207.1	Forward: TACTGCTGCGCTTTCTACCC<br>Reverse: TCTGTCGTATCCTCCCGTCA	qRT-PCR	139	involved in spermiogenesis [77]
armadillo repeat-containing protein 4-like (*armadillo4*)	XM_025228505.1<br>XM_025228495.1<br>XM_025228481.1<br>XM_025228490.1	Forward: ATTGAAGCGAGGCCACATGA<br>Reverse: ACGGTCCAAGATAAGCTGGC	qRT-PCR	117	involved in spermatid elongation and maturation [77]
meiotic recombination protein SPO11-like (*SPO11*)	XM_025253491.1<br>XM_025253492.1	Forward: CACAGGCTTTTGAGAACCACC<br>Reverse: CATGGTCTCAATCTGCTCGG	qRT-PCR	200	involved in the spermatogenic meiosis [78]
meiotic recombination protein REC8 homolog (*REC8*)	XM_025239316.1	Forward: TTCCTATGCCCATGCCATCC<br>Reverse: ATTCCGTTTCCGACATGCCT	qRT-PCR	129	involved in the spermatogenic meiosis [78]
boule-like (*Pcbol*)	XM_025239622.1	Forward: CCACAGATGTCTCCCCATCG<br>Reverse: GTATTTGCGGCGATTCCACC	qRT-PCR	118	

doublesex- and mab-3-related transcription factor 2-like (Dmrt2)	XM_025255755.1	Forward: ACACCAGGCGACAGAAATGT<br>Reverse: GCAAAGCTCGTAGATTCCGC	qRT-PCR	101	pivotal for normal testicular development and function [79]
SRY-related HMG box gene 2 (*Sox2*)	XM_025222813.1	Forward: CCAGTACGGTTACGCCATGT<br>Reverse: GGCGGCATACTGGTTCATCT	qRT-PCR	121	essential for proper development of spermatogonial stem cells [80]
green fluorescence protein (*GFP*)	MN443913.1	Forward: taatacgactcactatagggGCGAGGGCGATGCCACCTAC<br>Reverse: taatacgactcactatagggCACGCTGCCGTCCTCGATGT	dsRNA synthesis	431	
boule-like (*Pcbol*)	XM_025239622.1	Forward: taatacgactcactatagggTCATCCACAGATGTCTCCCCA<br>Reverse: taatacgactcactatagggAAACTCCAGCTCTGTCAGCAA	dsRNA synthesis	205	

Note: As for genes with multiple alternative splicing variants or isoforms, primers were designed based on their overlapped coding sequences and underlined. Lowercase letters denoted T7 promoters.

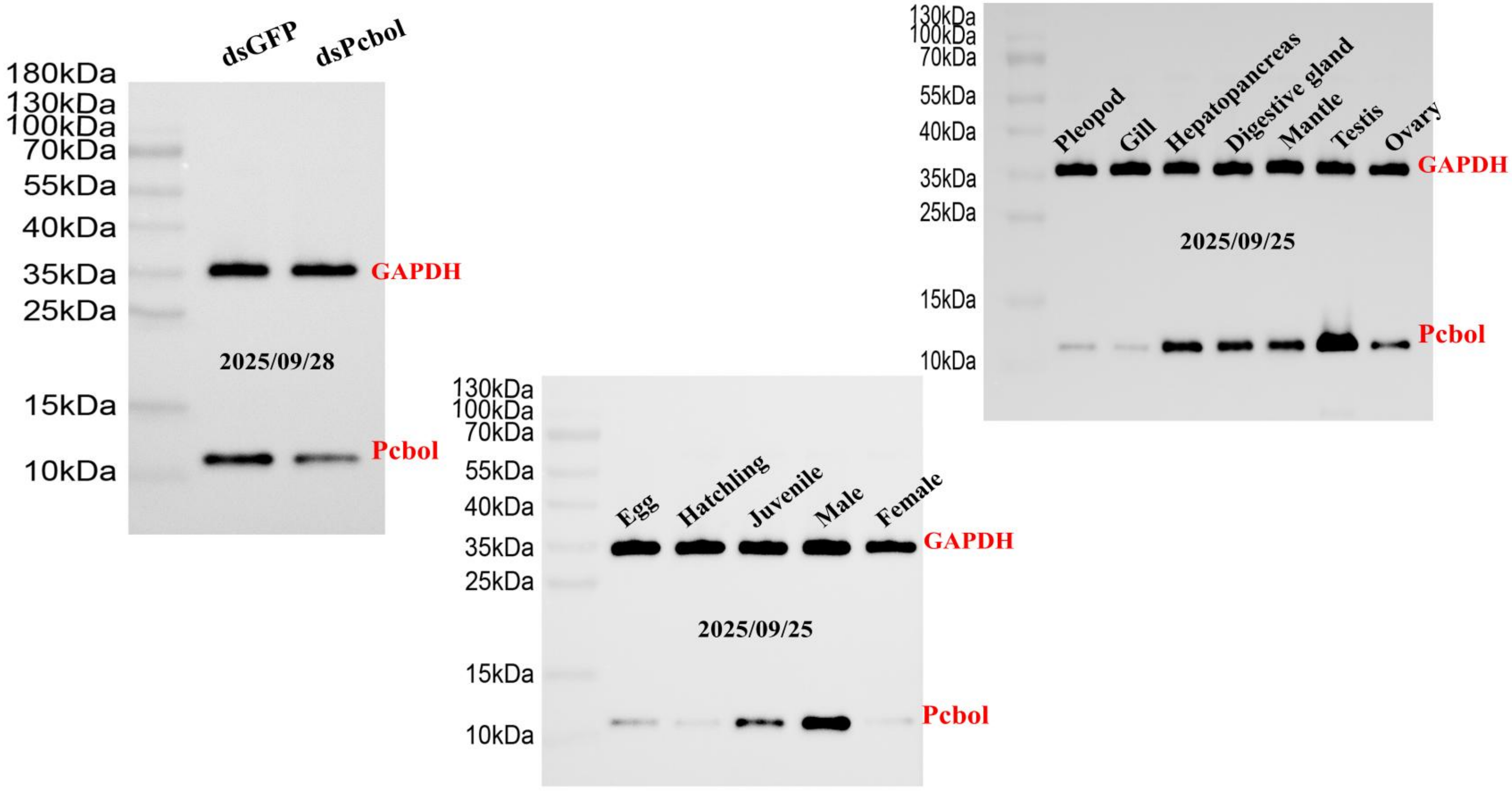

Figure S1. Original Western blotting images for Figure 3B (left), Figure 2A (middle) and Figure 2C (right).

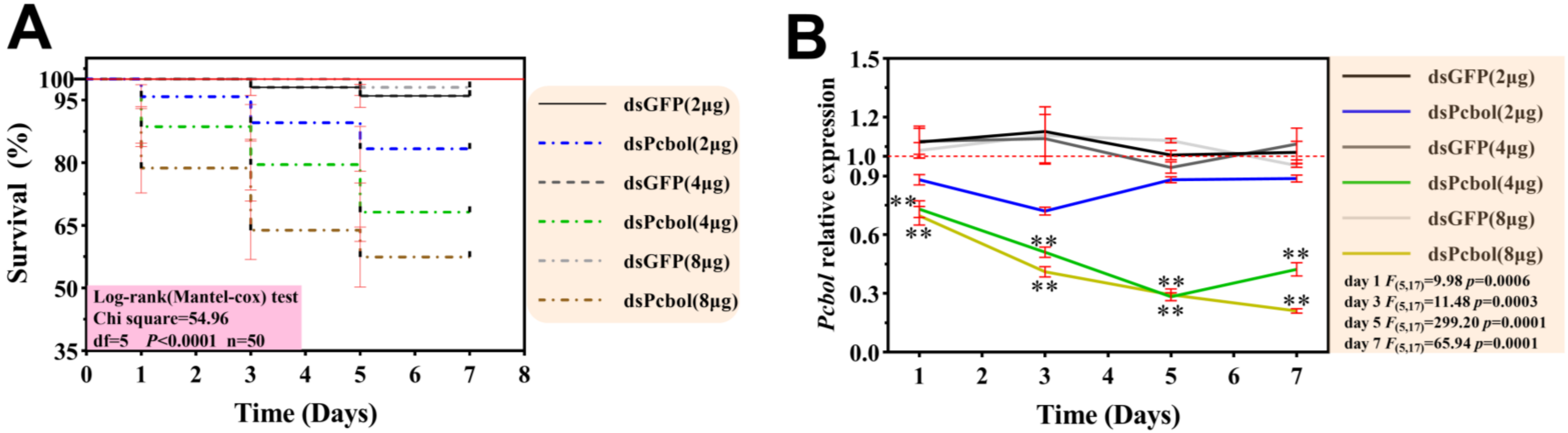

Figure S2. The survival rate of individual male (A) and *Pcbol* expressions of testes (B) subjected to different concentrations (2/4/6/8 μg) of dsRNA during multiple time periods (1/3/5/7 day). Significant differences in survival are assessed by logrank (Mantel-Cox) test in GraphPad Prism (n=50, p<0.0001). The mRNA levels of *Pcbol* are compared using one-way ANOVA followed by post-hoc Tukey's test, with asterisks indicative of significant differences relative to the control group (dsGFP) at the same concentration and time point (**p < 0.01). Data are presented as mean ± SEM (n=3).
